# Ability of device to collect bacteria from cough aerosols generated by adults with cystic fibrosis

**DOI:** 10.12688/f1000research.9251.1

**Published:** 2016-08-05

**Authors:** David N. Ku, Sarah K. Ku, Beth Helfman, Nael A. McCarty, Bernard J. Wolff, Jonas M. Winchell, Larry J. Anderson

**Affiliations:** 1 Georgia Institute of Technology, Atlanta, GA, 30332, USA; 2 MD Innovate, Inc, Decatur, GA, 30030, USA; 3 Emory Children’s Center for Cystic Fibrosis Research, Emory University, Atlanta, GA, 30322, USA; 4 Department of Pediatrics, Emory University, Atlanta, 30322, USA; 5 Respiratory Diseases Branch, Centers for Disease Control and Prevention, Atlanta, GA, 30333, USA; 6 Division of Infectious Diseases, Department of Pediatrics, Emory University and Children’s Healthcare of Atlanta, Atlanta, GA, 30322, USA

**Keywords:** Cystic fibrosis, aerosols, specimen collection, respiratory infections, etiology

## Abstract

**Background**: Identifying lung pathogens and acute spikes in lung counts remain a challenge in the treatment of patients with cystic fibrosis (CF). Bacteria from the deep lung may be sampled from aerosols produced during coughing.

**Methods**: A new device was used to collect and measure bacteria levels from cough aerosols of patients with CF. Sputum and oral specimens were also collected and measured for comparison. *Pseudomonas aeruginosa*, *Staphylococcus aureus*, *Klebsiella pneumoniae*, and *Streptococcus mitis* were detected in specimens using Real-Time Polymerase Chain Reaction (RT-PCR) molecular assays.

**Results**: Twenty adult patients with CF and 10 healthy controls participated. CF related bacteria (CFRB) were detected in 13/20 (65%) cough specimens versus 15/15 (100%) sputum specimens. Commensal *S. mitis* was present in 0/17 (0%, p=0.0002) cough specimens and 13/14 (93%) sputum samples. In normal controls, no bacteria were collected in cough specimens but 4/10 (40%) oral specimens were positive for CFRB.

**Conclusions**: Non-invasive cough aerosol collection may detect lower respiratory pathogens in CF patients, with similar specificity and sensitivity to rates detected by BAL, without contamination by oral CFRB or commensal bacteria.

## Introduction

The etiology of lower respiratory tract infections in the lungs is difficult to determine, in part because a good quality specimen from the site of the infection is not readily available ^1– 4^. Access to such a specimen would be an important advance in the monitoring and treatment of cystic fibrosis (CF), as well as other lower respiratory tract infections, such as pneumonia, tuberculosis, asthma, lung cancer, etc. Presently, oropharyngeal (OP), sputum, and bronchoalveolar lavage (BAL) specimens are typically used to monitor CF patients. OP specimens may be appropriate for detecting viruses, but are not ideal for most bacterial pathogens. Sputum is commonly collected to monitor CF but often contains contaminants and cystic fibrosis related bacteria (CFRB) from the upper respiratory tract. The difficulty some patients have in producing an acceptable sputum specimen further decreases the value of these samples, often causing the physician to treat the patient empirically ^1– 4^. BAL provides a specimen from the lungs but is an invasive procedure that cannot be routinely used. BAL specimens may also collect contaminants from the upper respiratory tract ^5– 7^.

**Figure 1.**
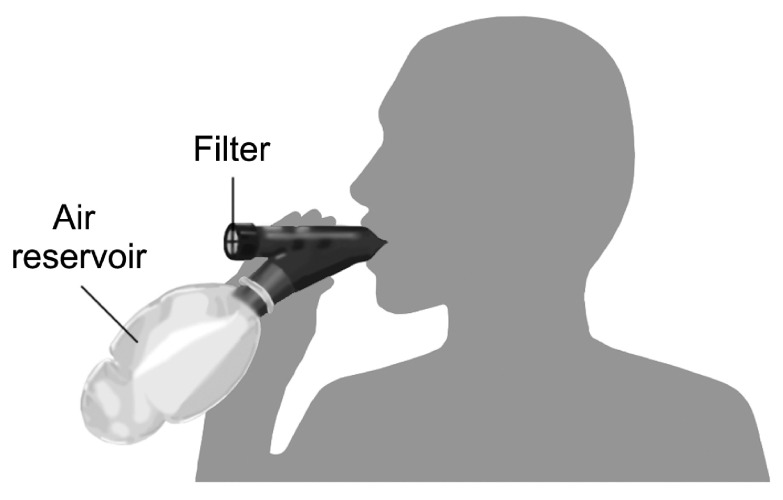
PneumoniaCheck™ specimen storage and transport container.

An alternative source for a lung specimen is from aerosols generated during coughs ^8– 12^. Studies show that one cough can generate as many as 66,000 expelled particles ^10, 13^. Patients that have lower respiratory tract infections can infect others through respiratory dispersion of pathogens in aerosols generated by coughing or sneezing. Coughing produces a higher concentration of pathogens from the lower lungs than normal exhalation or sneezing ^8– 13^. A new cough specimen collection device (PneumoniaCheck™, Figure 1) collects aerosols from the lungs onto a micropore filter while minimizing contamination from the upper respiratory tract. Microbiology or molecular assays can then be used to detect pathogens collected on the device’s filter.

**Figure 2.**
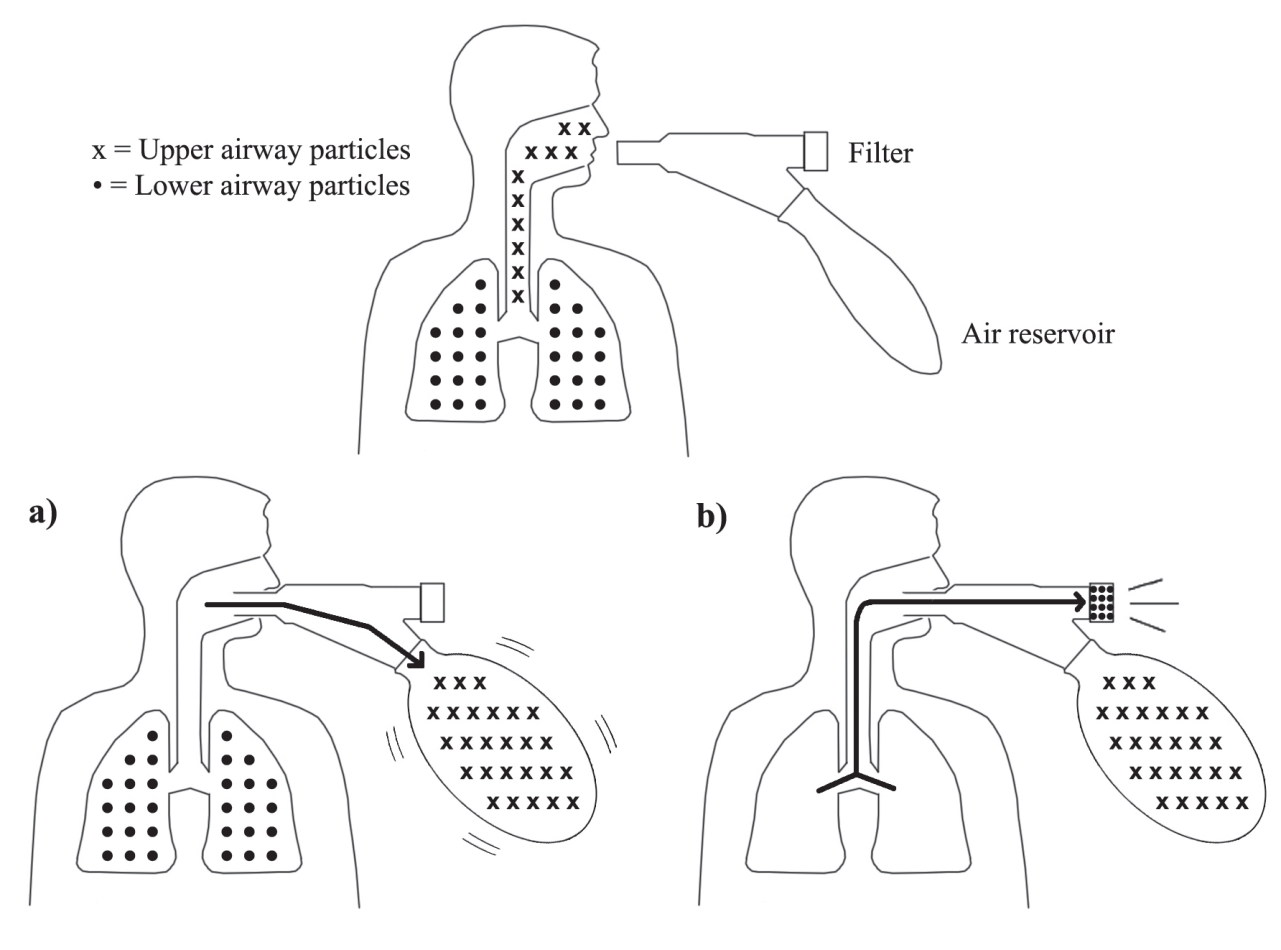
PneumoniaCheck™ fluid mechanics airway separation. ( **a**) Contaminated upper airway particles from the mouth initially fill up air reservoir. ( **b**) Then, uncontaminated lower airway particles from the lungs are captured onto filter.

The device uses a reservoir to separate oral contents from deep lung aerosols using fluid mechanics for separation ( Figure 2). The initial volume of air that comes from exhalation or coughing is contaminated air from the upper respiratory tract, also known as anatomic dead space. When a patient coughs into the device, this air from the upper airways first flows into the reservoir ( Figure 2a). The exhaled air flows to the reservoir first as it has the least resistance compared to the filter at the end of the device. This reservoir has a volume of 250 ml, approximately 100 ml greater than the volume of anatomic dead space in the average adult ^15^, which ensures that all of the upper airway aerosols are completely separated out. The expanded reservoir is inelastic, creating a back-pressure, so subsequent exhaled breath is forced through the microbial filter ( Figure 2b). Therefore, only lung aerosol contents are collected onto the filter and are free from upper airway contamination.

A previous study demonstrated that the device’s filter is >99% effective in collecting airborne bacteria (approximately 3.1 μm in diameter) and viruses (approximately 2.8 μm in diameter) ^16^. Sampling from normal individual controls showed zero collection of oral contents on the filter, even with up to 15 ml of liquid in the mouth (simulating sputum). The PneumoniaCheck™ device has been shown to significantly separate the lower airway gas from the upper airway gas based on oxygen and alcohol levels (p<0.0001) ^16^.

CF is a genetic disease that affects the lungs of approximately 28,000 children and adults in the United States each year ^17^. People with CF often have chronic lung infections and require regular monitoring to ensure that bacterial colonization does not develop into infection ^26, 27^. We used specimens from sputum and coughs to compare their abilities to capture, identify, and quantify relative levels of lung bacteria in adult CF patients. The goal of this study is to determine if the cough device can capture lung pathogens from adult patients with chronic lung infection while simultaneously excluding oral bacteria.

## Materials and methods

### Subjects

Patients with CF (n=20) aged >18 years old were recruited from the Emory Cystic Fibrosis Center Adult Clinic in Atlanta, Georgia. The Emory Institutional Review Board (H08353) approved the study and participants provided their written, informed consent. The sample size was sufficiently powered to demonstrate statistical significance for lower lung sampling without oral contamination. Tests of paired proportions were conducted using an exact form of the McNemar test to compare the presence of CFRB between two samples (i.e. cough and sputum). The Wilcoxon signed rank test was used to compare cycle threshold ( *C _T_*) values between the different methods of sampling. The *C _T_* value of 60 was used as the upper limit of detection for all PCR assays to determine relative quantity of bacteria in each specimen.

### Clinical measurements

Throat swabs and cough device specimens were collected from 10 healthy, non-smoking subjects for normal controls. Separately, a sputum specimen and cough device specimen were each collected from 20 adult patients with CF. Cough device specimen collection preceded sputum specimen collection in order to help induce sputum. Specimen collections were supervised and emergency equipment was readily available. *Streptococcus mitis* is a commensal bacterium that is found in the mouth but not in the lungs ^14^. *Streptococcus pneumoniae* and *Staphylococcus aureus* are also commonly found in the oral cavity ^3^. *Pseudomonas aeruginosa*, *Staphylococcus aureus* and *Klebsiella pneumoniae* are cystic fibrosis related bacteria (CFRB) ^18– 20^. Oral and cough specimens were analyzed for these bacteria to determine levels of oral contamination.

### Determining sufficient aerosol collection

Fennelly’s Cough Aerosol Sampling System (CASS) ^29, 30^ and Knibbs’ Distance Rig ^13^ have demonstrated that cough particles can carry substantial concentrations of bacteria from lower respiratory infections. A previous article on the cough collection device describes the ability of the device to selectively sample from the lower lungs while excluding oral contaminants ^16^. The cough device used in this study provides a less cumbersome option to Fennelly’s and Knibbs’ methods for lung specimen collection. Each patient coughed 10 times into the device to ensure sufficient aerosol collection.

### Microbiology

Microbiology culturing has several limitations that decrease the efficiency and effectiveness of rapid diagnosis ^24^. Throat, sputum, and cough specimens were all analyzed using molecular PCR methods. All specimens were processed in a BSL 2 safety cabinet. The cough device filter was removed, placed into a 2 ml sterile freezer vial, and stored at -80°C. Respiratory secretions captured on the filter were removed by hydrating the filter with 1mL of lysis buffer (MagNA Pure LC lysis buffer; Roche Applied Science, Indianapolis, IN), vortexing, incubating for 5 min at room temperature, and collected using a pipette. Fluid remaining in the filter was collected by placing the filter in a sterile Costar SpinX microfuge tube with a 0.45 micron filter (Corning Inc., Corning, NY), centrifuging for 1 min at 10,000 rpm, and retrieved using a pipette. The residual fluid was then combined with original collected fluid and then 400 μL was extracted on the MagNA Pure Compact Instrument (Roche Applied Science) per the manufacturer’s instructions. The extracted nucleic acid was eluted into 100 μL of elution buffer and stored at -80°C for qPCR testing.

The sputum specimen was mixed with 1 mL of phosphate buffered saline (PBS), homogenized with pipetting and vortexing, mixed with a 12.5 mM equal volume of freshly prepared dithiothreitol (DTT, No Weigh™ format, Fisher Scientific), and incubated at room temperature for 30 min with periodic vortexing. The resultant solution was divided into 400 μL aliquots and stored at -80°C. A 400 μL aliquot of the processed sample was then extracted on the MagNA Pure Compact Instrument and stored as described above.

The extracted nucleic acid was tested for *P. aeruginosa*, *S. aureus*, *K. pneumoniae*, and *S. mitis* targets by individual real-time PCR assays. The primer and probe sequences for these assays have been previously described ^25^. The *S. mitis* primers are: Forward TTTTGTCATCTAGCCTTGC; Reverse GCAGTCATATCATCACCTTC and Probe ACTTGGGCAATCCCGACAGATTCTAAC, with a 5' FAM reporter and a 3' BHQ quencher. The PCR reactions were done with 5 μl of extracted nucleic acid from the specimens plus 12.5 μl of PerfeCTa Multiplex qPCR SuperMix (catalog no. 95063-200; Quanta BioSciences), 0.5 μM final concentrations of each primer, 0.1 μM final concentration of the probe, and nuclease-free water (catalog no. P1193; Promega) to a final reaction volume of 25 μL. Real-time PCR reactions were performed using an ABI 7500 standard machine (Life Technologies, Carlsbad, CA) with enzyme activation at 95°C for 5 min, followed by 45 cycles of 95°C for 15 seconds and 60°C for 1 min. All specimens from CF patients were run in duplicate for each target. The *C _T_* values for individual PCR assays were used as an indication of the relative quantity of bacteria in the specimen.

## Results

All subjects completed specimen collection safely. Ten healthy subjects were used for controls. Sputum and cough specimens were successfully collected from 20 adult patients with CF, with the exception of five patients who could not produce a sputum specimen.

Normal controls demonstrated a high incidence of false positives from oral sampling, shown in Table 1. Bacteria were isolated from throat swabs in 4/10 (40%) normal, healthy control subjects. *S. pneumoniae* was positive in 2/10 (20%) oral specimens and *S. aureus* was positive in 3/10 (30%) oral specimens, with one subject positive for both bacteria. In contrast, 0/10 (0%, p=0.0313) cough specimens were positive for bacteria in normal controls. The calculated true negative rate or specificity for sputum specimens was 60% and 100% for cough specimens.

**Table 1.** Detection of bacteria in throat and cough specimens from normal, healthy controls.

	Throat	Cough
1	*S. aureus*	Negative
2	Negative	Negative
3	Negative	Negative
4	*S. aureus*	Negative
5	*S. aureus*, *S. pneumoniae*	Negative
6	Negative	Negative
7	Negative	Negative
8	*S. pneumoniae*	Negative
9	Negative	Negative
10	Negative	Negative

Specificity in the CF patients was similar. For the CF patients, *S. mitis* was isolated from 13/14 (93%) sputum specimens but in none of the cough specimens (0%, p=0.0002). The cough specimens collected no *S. mitis*. CFRB was collected in both specimen types. *P. aeruginosa* was isolated from 13/15 (87%) sputum specimens and 9/20 (45%) cough specimens (p=0.0213). *S. aureus* was isolated from 9/15 (60%) sputum specimens and 3/20 (15%) cough specimens. *K. pneumoniae* was isolated from 2/15 (13%) sputum specimens and 3/20 (15%) cough specimens.

In aggregate, sputum specimens were positive for CFRB in 15/15 (100%) samples. Cough specimens were positive for CFRB in 13/20 (65%) samples. The sputum specimens had a 93% rate of oral commensals. Sputum specimens were positive for three or more pathogens in 2/15 (13%) samples, and positive for two or more pathogens in 7/15 (47%) samples. In contrast, cough specimens had no commensals and were positive in 65% of the CF patients. The cough specimens were positive for two or more pathogens in 2/20 (10%, p<0.05) specimens and no cough specimens were positive for three or more pathogens. The results of these real-time PCR identifications are listed in Table 2.

**Table 2.** Detection of bacteria in sputum and cough specimens from adult CF patients.

	Sputum			Cough		
	n=15*	%	*C _T_* range	n=20	%	*C _T_* range
***P. aeruginosa***	13/15	87%	18–33	2/20	45%	33–42
***S. aureus***	9/15	60%	24–38	3/20	15%	36–40
***K. pneumoniae***	2/15	13%	37–38	3/20	15%	39–43
**CFRB total**	15/15	100%	18–38	13/20	65%	33–43
**2+ pathogens**	7/15	47%	18–38	2/20	10%	36–43
**3+ pathogens**	2/15	13%	18–38	0/20	0%	N/A
***S. mitis***	13/14†	93%	24–41	0/17§	0%	N/A

* Five patients were unable to produce viable sputum specimens † Six sputum specimens were not tested for *S. mitis* § Three cough specimens were not tested for *S. mitis*

*C _T_* values are inversely proportional to the quantity of bacteria in a sample, i.e. small values indicate higher quantities of colony forming units (CFU). For the CFRB samples, *P. aeruginosa C _T_* values ranged from 18–33 in sputum specimens and 33–42 in cough specimens. *S. aureus C _T_* values ranged from 24–38 in sputum specimens and 36–40 in cough specimens. For both *P. aeruginosa* and *S. aureus* the cough and sputum specimens significantly differed in *C _T_* values (p=0.0017 and 0.0092, respectively). *K. pneumoniae C _T_* values ranged from 37–38 in sputum specimens and 39–43 in cough specimens. Thus, the *C _T_* values for cough specimens were consistently higher than those of sputum. Note that the cough filter samples from normal controls exhibited no pathogens up to *C _T_* values of 60.

## Discussion

It is widely recognized that a simple, safe, non-invasive, low maintenance, inexpensive, widely accessible sampler is needed for the collection of lower respiratory pathogens ^34– 37^.

Collection of lower lung contents by coughing is much easier than BAL specimen collection. The method is convenient for patients who are already inclined to cough and they reported that the use of the device helped clear their lungs. Cough specimens may provide a non-invasive yet specific sample for in-home surveillance to watch for spikes in lung pathogens in patients with CF. Use of the device to collect cough aerosols has the potential to provide a clean alternative to oral samples for detecting lower lung pathogens.

As is well known, commonly used samples of sputum or OP swabs show strong contamination in the upper airway ^3, 4^. Nearly all of the sputum specimens were positive for the oral commensal *S. mitis*. In contrast, none of the cough specimens were positive for *S. mitis*. This difference in the commensal *S. mitis* between sputum and cough specimens reiterates the unreliability and low specificity of sputum ^3, 4^. Further, not all patients can produce an adequate sputum sample. Examining the 12 subjects with paired sputum and *S. mitis* results, 8/12 (67%) sputum and cough specimens had concordant positives, although six of these eight were positive for additional bacteria in sputum. These six sputum specimens with multiple bacteria likely indicate false positives from oral contamination rather than co-infection.

The number of concordant sputum and cough specimens (2/12, 17%) was small. Conversely, the pathogen detected in the cough specimen was different from that observed in the sputum specimen. 2/12 (17%) sputum specimens did not identify bacteria that were identified in cough specimens, possibly reflecting masked readings associated with commensal distraction. The differences demonstrate that the cough device is not just collecting sputum.

*P. aeruginosa* is the most common bacterium found in lungs of adult CF patients ^19^, and was also the most prevalent bacterium collected in our cohort. 13/20 (65%) cough specimens were positive for CFRB. This incidence and distribution of pathogens in CF is similar to the 59% positive for CFRB in BAL sampling ^22, 23^. Prior series of BAL specimens in similar populations have yielded positive CFRB of 59–85%, similar to the 65% positivity from the cough device illustrating comparable sensitivity ^22, 23^.

Collection of exhaled aerosols has been studied by several previous groups. An alternate device for aerosol collection is the RTube™; however, it varies greatly in design and function ^28^. The RTube™ system is designed to collect from all exhaled breath that condenses ^28^ while PneumoniaCheck™ is designed to collect particulate sized lung aerosols and separate out the mouth contents ^16^. The majority of exhaled gas passes out of the end of the RTube™ as only water condensate is intended to be collected. Exhaled breath condensate can be a useful specimen for identifying pH levels, but is generally not viewed as a reliable specimen for identifying lower respiratory infections ^34– 37^.

Wainwright *et al*., reported detecting *P. aeruginosa* in cough aerosols by culture ^12^. They reported 25/28 (89%) positive in a mixed population of children and adults with CF using a cough aerosol sampling system (CASS) for 5 minutes with each subject ^12^. Similarly, Knibbs *et al.* reported that 14/18 (78%) patients aerosolized *P. aeruginosa* that remained viable and presumably transmissive up to 45 minutes after coughs sampled on an Anderson impactor ^13^. Knibbs *et al*. used conventional microbiology cultures to quantify colony forming units. Both of these studies used a specially constructed aerosol sampler that is expensive, cumbersome, and difficult to use in a clinical setting. For these studies, patients cough into a standard mouthpiece and aerosols are sucked into impactors using vacuum air pumps. The CASS system was not designed for routine use in clinical settings and the mouthpiece was not designed to exclude oral contents. These designs differ from PneumoniaCheck™, which has a mouthpiece designed to specifically exclude oral contaminants ^16^. The high incidence of *P. aeruginosa*, using the snorkel type mouthpiece and tubing, may reflect some collection of oral contents using CASS.

RT-PCR may be used to quantify the amount of pathogens in a sample. As more material is collected on the filter, the *C _T_* counts will drop similar to the inverse of CFUs ^32^. It should be noted that an aerosolized lung specimen should have higher *C _T_* values compared to the liquid specimens of sputum due to a lack of contamination and the small physical volume of aerosols. *C _T_* values in the sputum specimens ranged from 19–38, whereas the range in cough specimens was 33–43 (p<0.001, Table 3). Nonetheless, *C _T_* values in all positive cough specimens are significantly lower than the baseline of >60 for normal controls. While the *C _T_* values are higher for the cough specimens, the background noise level of the virgin filter is >60, allowing limits of detection by PCR that may be more sensitive to lung CFRB.

**Table 3.** *C _T_* values of sputum and cough specimens grouped by pathogen.

Subject	*P. aeruginosa*		*S. aureus*		*K. pneumoniae*		*S. mitis*	
#	Sputum	Cough	Sputum	Cough	Sputum	Cough	Sputum	Cough
1	22	33	-	-	-	-	41	-
2	N/A	42	N/A	-	N/A	-	N/A	-
3	32	-	-	-	-	-	32	-
4	21	41	36	-	-	-	24	-
5	N/A	-	N/A	-	N/A	-	N/A	-
6	18	-	-	-	-	41	32	-
7	N/A	-	N/A	-	N/A	-	N/A	-
8	N/A	35	N/A	-	N/A	-	N/A	-
9	N/A	-	N/A	36	N/A	43	31	-
10	19	-	38	40	37	-	N/A	N/A
11	19	42	27	-	38	-	N/A	N/A
12	20	-	-	-	-	-	30	-
13	33	39	30	-	-	-	33	-
14	33	40	24	-	-	-	33	-
15	-	-	37	-	-	-	32	-
16	25	-	-	-	-	-	31	-
17	-	-	33	-	-	39	27	-
18	25	-	-	-	-	-	29	-
19	25	39	29	39	-	-	29	-
20	22	42	34	-	-	-	-	N/A

One can utilize the *C _T_* values to compare relative amounts of pathogens being coughed by an individual patient compared with a population ^32, 33^. Jones-López *et al.* found that both CF and TB patients can produce aerosols with viable pathogens, but the amount of pathogens produced by individuals varies greatly ^31^. Patients with high amounts of *M. tuberculosis* in cough aerosols were more likely to have transmitted to others ^30^. Those that produce large amounts of pathogens in coughs may be more efficient transmitters, e.g. “superspreaders” ^13, 30^. The quantity of pathogens in a cough may be a critical metric in transmission of infectious disease, controlling epidemics, and monitoring colonization. In the Jones-López *et al*. study, the amount of aerosolized *M. tuberculosis* fell dramatically after three weeks of treatment. Therefore, a cough specimen could also be used to monitor levels of resistant bacteria, if present.

Published guidelines for CF patients suggest acquiring quarterly respiratory specimens to monitor lung infections ^26, 27^. Cough specimens may be a more specific and sensitive method for monitoring colonization and determining infectivity. Additional studies could explore this application further by comparing *C _T_* values with symptoms. If a CF patient is monitored on a regular basis using cough specimens, a sudden decrease in *C _T_* value may indicate a change in pathogen burden ^32, 33^. The *C _T_* value of pathogen burden in cough aerosols may be useful as a measurement to determine if the lung burden is growing.

This study has several limitations. Our study included only adult patients; thus, we cannot comment on the aerosol production during coughing by pediatric patients. This study reports on 20 patients. Most studies in the literature have a similar number of subjects, since lower respiratory identification has always been an enormous challenge ^12, 13^. Future studies may evaluate the benefits of requiring more coughs or coughing for a specified amount of time, such as 5 minutes, to establish *C _T_* thresholds for this new method of specimen collection. The RT-PCR molecular assays used in this study are not available at all hospitals, although a few commercial laboratories can provide clinical respiratory identification services.

## Conclusion

In summary, we have shown that a new device can collect lung pathogens from adult patients with CF from cough aerosols with identification using molecular assays. The device excludes oral contaminants showing higher specificity than sputum samples. Identifying causative pathogens in the lower respiratory tract is likely to play a significant role in patient management ^24^. The data in this study suggest an alternative to sputum collection for the identification of lower respiratory pathogens.

## Consent

Written informed consent was obtained by all participants through Institutional Review Board Protocol #000-2492 approved by Georgia Institute of Technology, Emory University, and US Centers for Disease Control and Prevention.

## Data availability

The data referenced by this article are under copyright with the following copyright statement: Copyright: © 2016 Ku DN et al.

All raw data are provided in the tables above.
