# Supplementary material for: Ability of device to collect bacteria from cough aerosols generated by adults with cystic fibrosis
Source: F1000Res. 2016 Aug 5;5:1920. [Version 1] doi: 10.12688/f1000research.9251.1 (PMC5054809; doi:10.12688/f1000research.9251.1)
Supplement: Peer review history

# Peer review: Referee response for version 1

**DOI:** 10.5256/f1000research.9959.r16745

**Published:** 2016-10-03

**Authors:** David E Griffith

**Affiliations:** 1 Division of Infectious Diseases & Global Medicine, Department of Medicine, Comprehensive Heart and Lung Disease, The University of Texas Health Center at Tyler, Tyler, TX, USA

Comment #1: I am not familiar with the term Cycle Threshold, “ *C _T_*”. The term is introduced in the first paragraph of the Methods and then mentioned again in the last paragraph of the Methods. It would have been helpful to me to have had a brief discussion of this term in the Introduction or Methods. The *C _T_* values were discussed in full paragraphs in the Results and Discussion sections so it is clearly an important metric for this study and there could be other readers not familiar with *C _T_*.

 Comment #2: Evaluation of microbiologic results in bronchiectasis patients is difficult. First, there is no “gold standard” test for identifying potential respiratory pathogens in the lungs of these patients. Microbiome studies suggest that a large number of potential respiratory pathogens populate the lungs of these patients but do not help determine which one(s) are responsible for symptoms or clinical deterioration and therefore might benefit from therapy. Similarly, microbiology results from BAL do not necessarily identify pathogens responsible for symptoms and clinical deterioration. I think these observations are pertinent with regard to the sensitivity and specificity claims of the authors. In Table 1, it is apparent that mouth flora is not sampled with the cough technique avoiding an important mechanism of specimen contamination. In Table 2, however, there is poor concordance between sputum and cough with regard to *Pseudomonas* and *Staph*, with these 2 potential pathogens isolated more commonly with sputum than cough. One interpretation of that observation is that sputum is more sensitive than cough for recovering potential respiratory pathogens in CF. The more frequent isolation of multiple respiratory pathogens with sputum could be interpreted the same way especially in light of the microbiome data. I am unsure where the authors believe the source of the “excess” *Pseudomonas* and *Staph* (as well as the specimens with multiple pathogens) is for sputum patients?  Are they suggesting these “excess” isolates are “contaminants”?

 Comment #3: I think the authors have convincingly shown that the PneumoniaCheck™ device avoids upper airway bacterial contamination during specimen collection with cough in CF patients. I think their findings with regard to the number and type of CF related respiratory pathogens and the clinical significance of those pathogens remains to be elucidated.

I have read this submission. I believe that I have an appropriate level of expertise to confirm that it is of an acceptable scientific standard.

# Peer review: Referee response for version 1

**DOI:** 10.5256/f1000research.9959.r16100

**Published:** 2016-09-21

**Authors:** Mats Kalin

**Affiliations:** 1 Department of Infectious Diseases, Karolinska Institutet, Stockholm, Sweden

Ku *et al, *describe the experiences with a newly designed device for collecting cough specimens from patients with CF. The device is constructed with the intention that dead space air, presumably containing a high concentration of oral contaminants, should be collected separately, while on the other hand cough material from the lower lungs is to be collected on a specific filter constructed so that bacteria should be trapped in such a way that the material may be used for RT-PCR.

 Title, abstract, methods and material are clearly described as is results. Discussion is adequate and relevant.

 The presented results indicate high specificity with low risk of oral contaminants in cough specimens than in sputum from 20 adult CF individuals. Actually only 12 patients produced a sputum specimen, so it is a small study. However the differences were significant.

 Sensitivity cannot be assessed with the way the study was carried out, but comparison with other studies indicate satisfactory results. Thus, the device seems to permit improved analysis of quantitative bacteriology in lower lung specimens from CF patients. The device is described as simple to use and is suggested to be used to follow lower respiratory tract microbiology in CF patients, so that increased concentrations of significant bacterial pathogens may be noted. This may be a step forward for the management of these patients. Further studies, including pediatric studies, are needed to corroborate the findings in this study and to explore the advantages of longitudinal follow up of CF patients

 PCR may not suffice all the time, since bacterial resistance may have to be detected and specified in order to find an adequate treatment alternative. Needless to say this is an increasing problem.

I have read this submission. I believe that I have an appropriate level of expertise to confirm that it is of an acceptable scientific standard.
